# Supplementary material for: Phages-bacteria interactions underlying the dynamics of polyhydroxyalkanoate-producing mixed microbial cultures via meta-omics study
Source: mSystems. 2025 Mar 28;10(4):e00200-25. doi: 10.1128/msystems.00200-25 (PMC12013262; doi:10.1128/msystems.00200-25)
Supplement: Supplemental material — Supplemental figures and information. [file msystems.00200-25-s0001.pdf]

Supporting Information for:

Phages-bacteria interactions underlying the dynamics of polyhydroxyalkanoates-producing mixed microbial cultures via meta-omics study

Jian Yao[1,2], Yan Zeng[1,2], Xia Hong[3], Meng Wang[4], Quan Zhang[4], Yating Chen[2,5], Min Gou[1,2,6], Zi-Yuan Xia[1,2], Yue-Qin Tang[1,2,6,*]

[1]College of Architecture and Environment, Sichuan University, Chengdu, Sichuan, China

[2]Sichuan Environmental Protection Key Laboratory of Organic Wastes Valorization, Chengdu, Sichuan, China

[3]Sinopec Shanghai Engineering Co. Ltd., Shanghai, China

[4]Sinopec (Dalian) Research Institute of Petroleum and Petrochemicals Co. Ltd., Dalian, Liaoning, China

[5]Institute for Disaster Management and Reconstruction, Sichuan University, Chengdu, Sichuan 610207, China

[6]Engineering Research Centre of Alternative Energy Materials and Devices, Ministry of Education, Chengdu, Sichuan, China

*Correspondence: Yue-Qin Tang

E-mail: tangyq@scu.edu.cn

Legends

Supplementary information

SI1: Synthetic wastewater composition and reactor operation strategy

SI2: The detailed determination method of PHA

SI3: Details of library construction and sequencing

Supplementary figures

Figure S1: The phyla of prokaryotic and phage communities in each reactor

Figure S2: The detailed information of bacterial MAGs in Reactor_A

Figure S3: The detailed information of bacterial MAGs in Reactor_B

Figure S4: The detailed information of bacterial MAGs in Reactor_C

Figure S5: The detailed information of bacterial MAGs in Reactor_D

Figure S6: The detailed information of bacterial MAGs in Reactor_E

Figure S7: The mean rank shift of pOTUs in each community

Figure S8: Community-level responses to viral interactions through time as recorded by CRISPR-Cas loci within the microbial community

Figure S9: The relationship between the number of linkages and the abundance

Figure S10: The linkages between phages and hosts on different days and the antiviral systems of hosts (Reactor-A)

Figure S11: The linkages between phages and hosts on different days and the antiviral systems of hosts (Reactor-B)

Figure S12: The linkages between phages and hosts on different days and the antiviral systems of hosts (Reactor-C)

Figure S13: The linkages between phages and hosts on different days and the antiviral systems of hosts (Reactor-D)

Figure S14: The linkages between phages and hosts on different days and the antiviral systems of hosts (Reactor-E)

Figure S15: The number of main AMGs in different metabolic pathways

Figure S16: Sequence similarity between phaCs

Figure S17: Sequence similarity between fadDs

Figure S18: Sequence similarity between phbBs

Figure S19: Sequence similarity between acdABs

Figure S20: Sequence similarity between fadJs

Figure S21: Sequence similarity between acs

Figure S22: Sequence similarity between ackAs

Figure S23: Schematic diagram of SBRs

Figure S24: The abundance curve of abundant OTUs in each MMC

Supplementary information

Supplementary information S1:

Carbon source concentrations of different synthetic wastewater used for reactors A, B, C, D, and E were acetate (3.42 g/L), propionate (2.67 g/L), butyrate (2.29 g/L), valerate (1.7 g/L) and lactate (3.11 g/L), respectively. The content of inorganic and trace element in these synthetic wastewaters was consistent, NH_4Cl (183.4 mg/L), $MgSO_4·7H_20$ (670 mg/L), EDTA (110 mg/L), $CaCl_2·2H_20$ (80 mg/L), K_2HPO_4 (48 mg/L), KH_2PO_4 (38 mg/L), $FeCl_3·6H_20$ (1.5 mg/L), H_3BO_3 (0.15 mg/L), $CuSO_4·5H_20$ (0.03 mg/L), KI (0.03 mg/L), $MnCl_2·4H_20$ (0.12 mg/L), $Na_2MoO_4·2H_20$ (0.06 mg/L), $ZnSO_4·7H_20$ (0.12 mg/L), $CoCl_2·6H_20$ (0.15 mg/L) and thiourea (10 mg/L). Thiourea was added to inhibit nitrification. The pH of synthetic wastewater was adjusted to 7.0±0.1.

The SBR reactor consists of an aeration system, a substrate transfer pumping system and a stirrer (Figure S1). In the feeding phase of a cycle, 1 L of fresh synthetic wastewater is pumped into the SBR. The air pump then pumps air through the aerator into the SBR to maintain fermentation broth dissolved oxygen (DO) above 4 mg/L. During the first approximately 1 hour of aeration, the SBR is in the feast stage (carbon source is still present) and the DO concentration is maintained at a low level. As the carbon source is depleted and the SBR enters the subsequent famine phase, the DO concentration suddenly rises to high levels. After 650 minutes of aeration, the aeration was stopped and the sludge in the SBR began to settle, which took 50 minutes. Finally, 1L of the supernatant was discharged from the SBR. The next step is to start a new cycle of the above.

Supplementary information S2:

The fermentation broth was collected in a 10 ml centrifuge tube and centrifuged at 15°C, 6000 rpm for 5 min, the supernatant was removed and the biomass sample was retained. The sludge samples were frozen at -80°C for 24h and then lyophilized in a vacuum

freeze dryer for 24h. The lyophilized sample was placed in a pressure-resistant hermetically sealed digestion tube and reacted with 2 ml of chloroform and 2 ml of esterification solution (1 L of methanol containing 1 g of benzoic acid and 30 ml of sulfuric acid) for 6 h at 105°C. After the reaction, the solution was cooled to room temperature, 1 ml of distilled water was added and shaken vigorously, and the lower organic phase was stratified and analyzed by GC-MS (QP2010SE, SHIMADZU, Japan). The chromatographic column was SHIMADZU SH-Rxi-5ms (30m×0.25mm×0.25μm). The specific parameters were as follows: high purity helium as carrier gas, 1.2 ml/min; inlet temperature of 250°C; column oven heating program: initial temperature of 40°C, followed by 10°C/min to 280°C and maintained for 5 min; mass spectrometry interface temperature of 280°C; quaternary rod temperature of 120°C; ion source temperature of 250°C; solvent delay time of 2.5 min; and full scan mode (m/z: 40-600. z: 40-600) was used.) Benzoic acid was used as the internal standard to quantify the PHA (PHB+PHV) content in the samples by using the peak area ratio and content of P(3HB-co-3HV) standard (sigma, 3HB:3HV = 91:9, m/m) to benzoic acid as the standard curve.

Supplementary information S3:

Extracted DNA was fragmented to an average size of about 400 bp using Covaris M220 (Gene Company Limited, China) for paired-end library construction. The paired-end library was constructed using NEXTFLEX Rapid DNA-Seq (Bioo Scientific, Austin, TX, USA). Adapters containing the full complement of sequencing primer hybridization sites were ligated to the blunt-end of fragments. Paired-end sequencing was performed on Illumina NovaSeq (Illumina Inc., San Diego, CA, USA) at Majorbio Bio-Pharm Technology Co., Ltd. (Shanghai, China) using NovaSeq 6000 S4 Reagent Kit v1.5 (300 cycles) according to the manufacturer’s instructions (www.illumina.com).

The RNA was subjected to standard Illumina library preparation with an Illumina® Stranded mRNA Prep, Ligation (Illumina, San Diego, CA, USA), and rRNA was depleted using a RiboCop rRNA Depletion Kit for Mixed Bacterial Samples (Lexogen, USA). Sequencing was performed on an Illumina Novaseq6000 sequencer (Illumina, San Diego, CA, USA) with a paired-end sequencing method at Majorbio Bio-Pharm

Technology Co., Ltd. (Shanghai, China).

Supplementary figures:

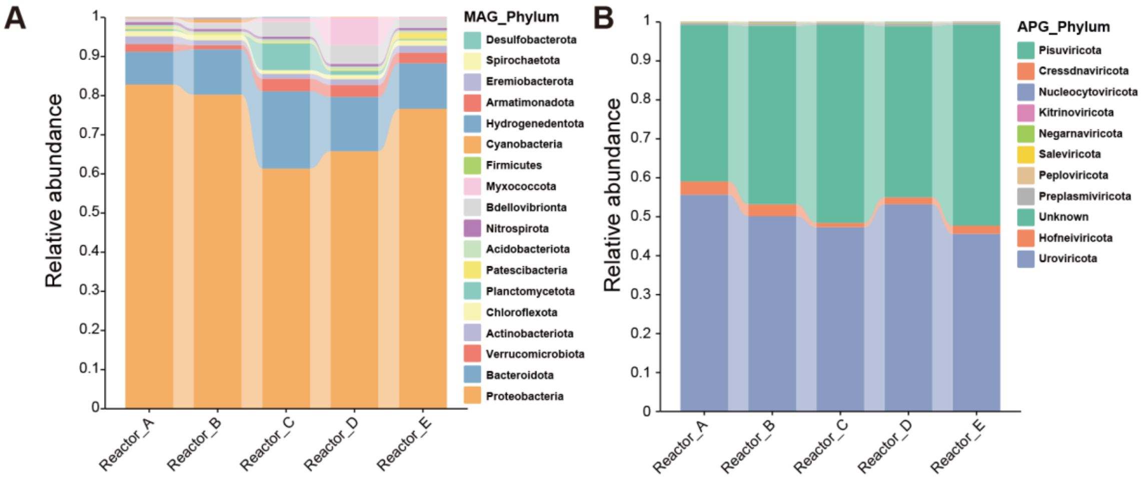

Figure S1 The phyla of prokaryotic and phage communities in each reactor

(A) The phyla in prokaryotic communities. (B) The phyla in phage communities.

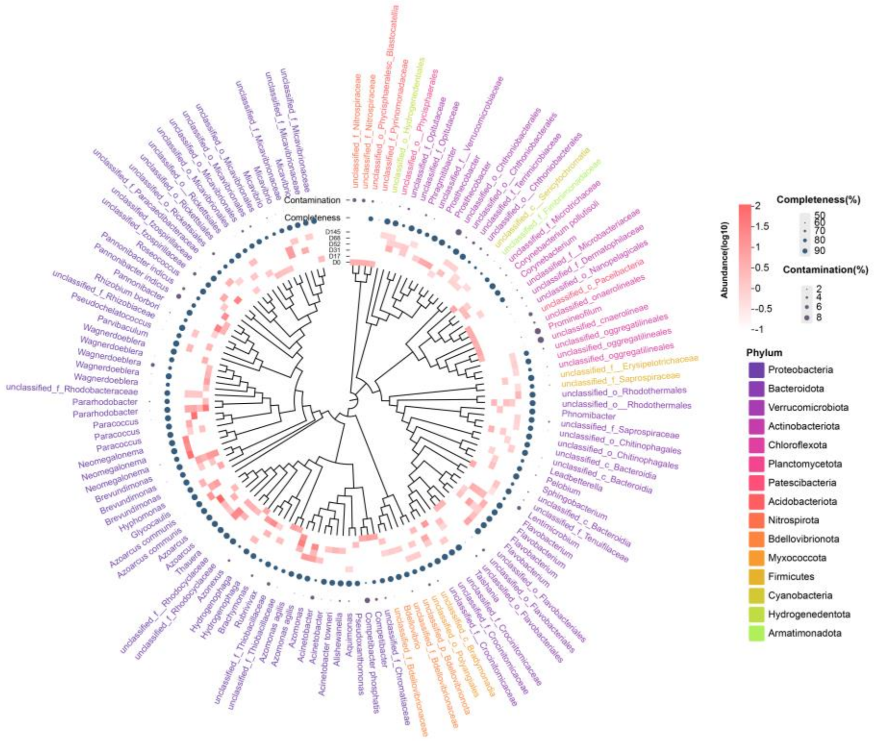

Figure S2 The detailed information of bacterial MAGs in Reactor_A

The circles from inside to outside are, in order, phylogenetic relationships, abundance at different times, completeness, contamination, and the genus of MAGs.

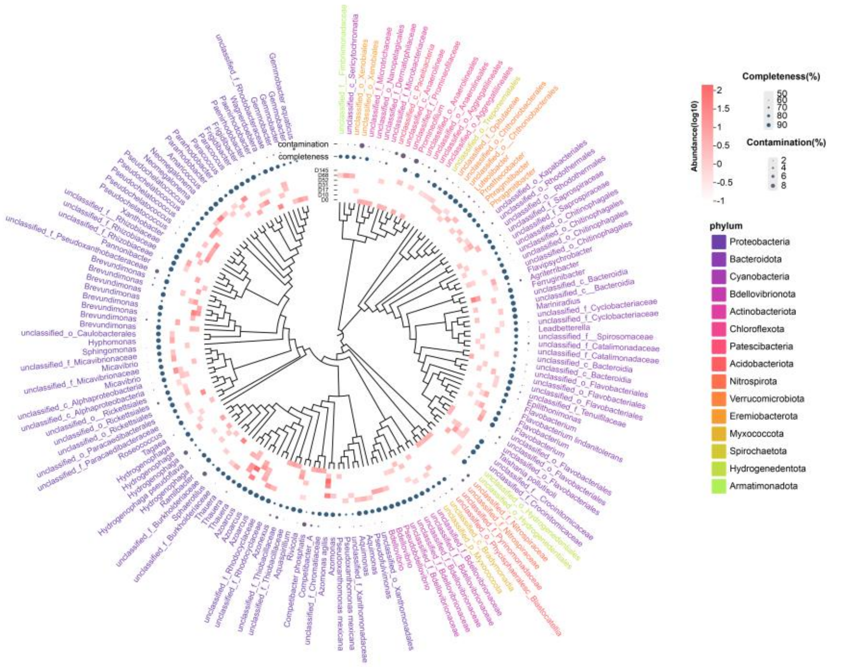

Figure S3 The detailed information of bacterial MAGs in Reactor_B

The circles from inside to outside are, in order, phylogenetic relationships, abundance at different times, completeness, contamination, and the genus of MAGs.

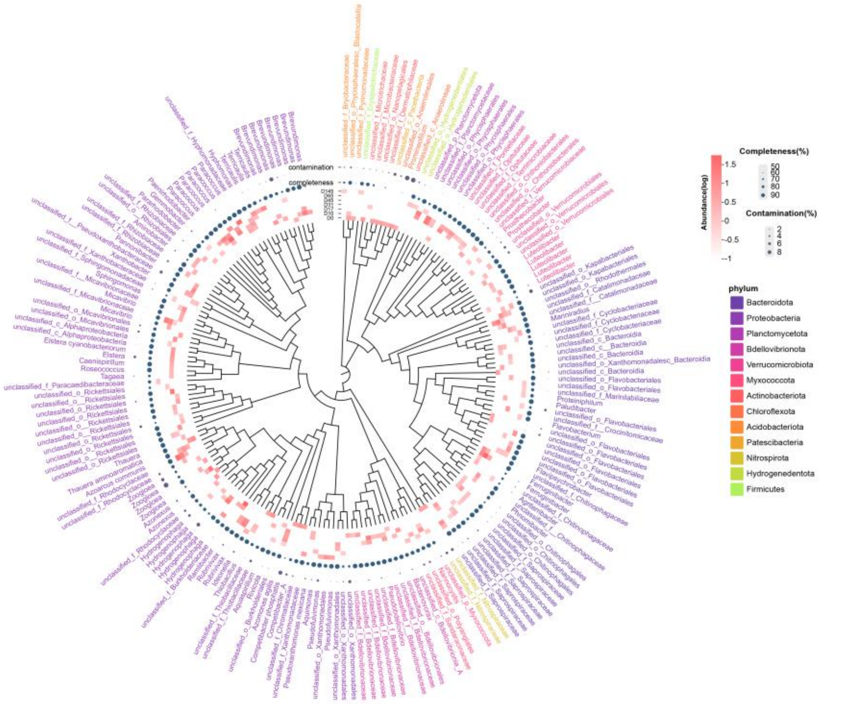

Figure S4 The detailed information of bacterial MAGs in Reactor_C

The circles from inside to outside are, in order, phylogenetic relationships, abundance at different times, completeness, contamination, and the genus of MAGs.

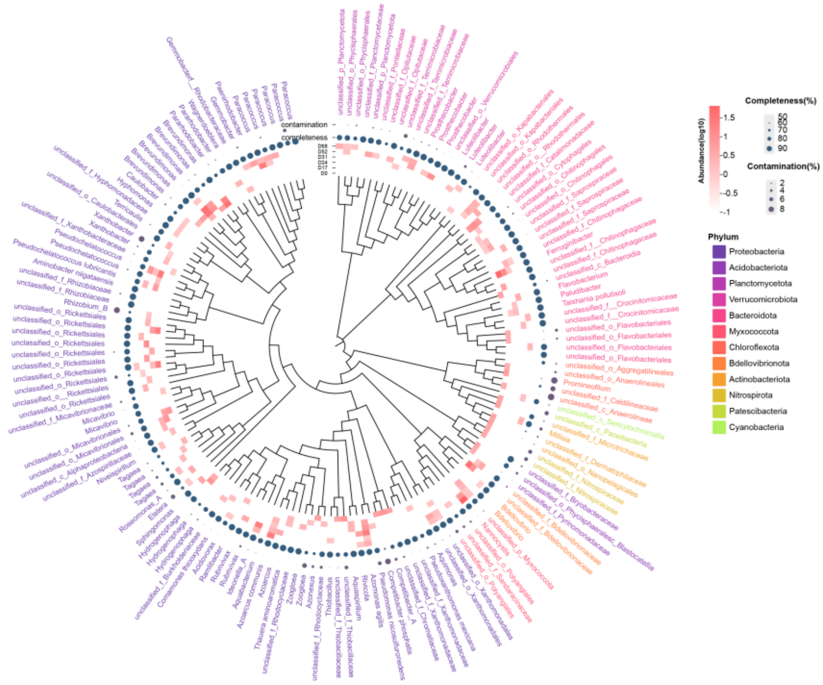

Figure S5 The detailed information of bacterial MAGs in Reactor_D

The circles from inside to outside are, in order, phylogenetic relationships, abundance at different times, completeness, contamination, and the genus of MAGs.

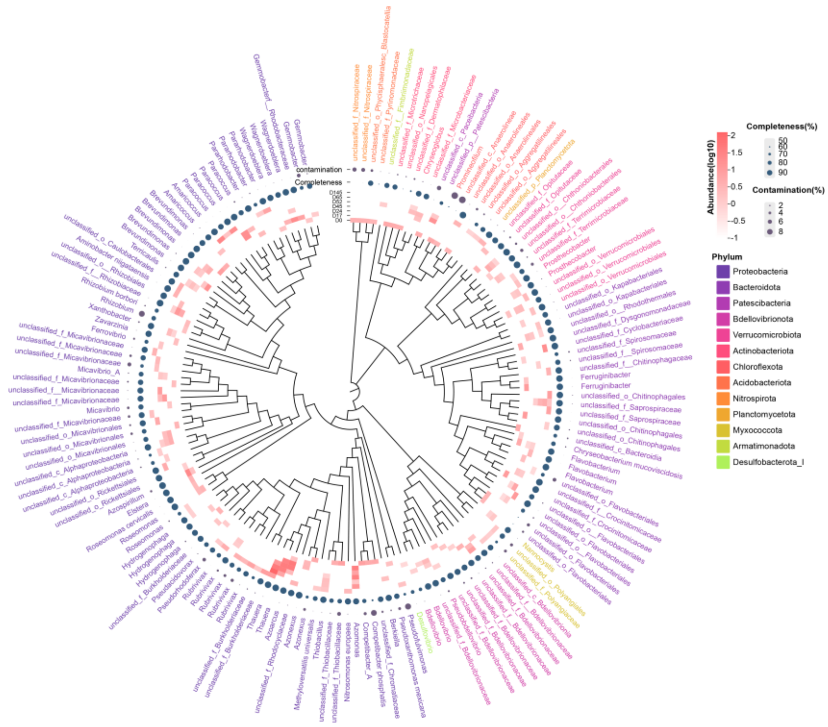

Figure S6 The detailed information of bacterial MAGs in Reactor_E

The circles from inside to outside are, in order, phylogenetic relationships, abundance at different times, completeness, contamination, and the genus of MAGs.

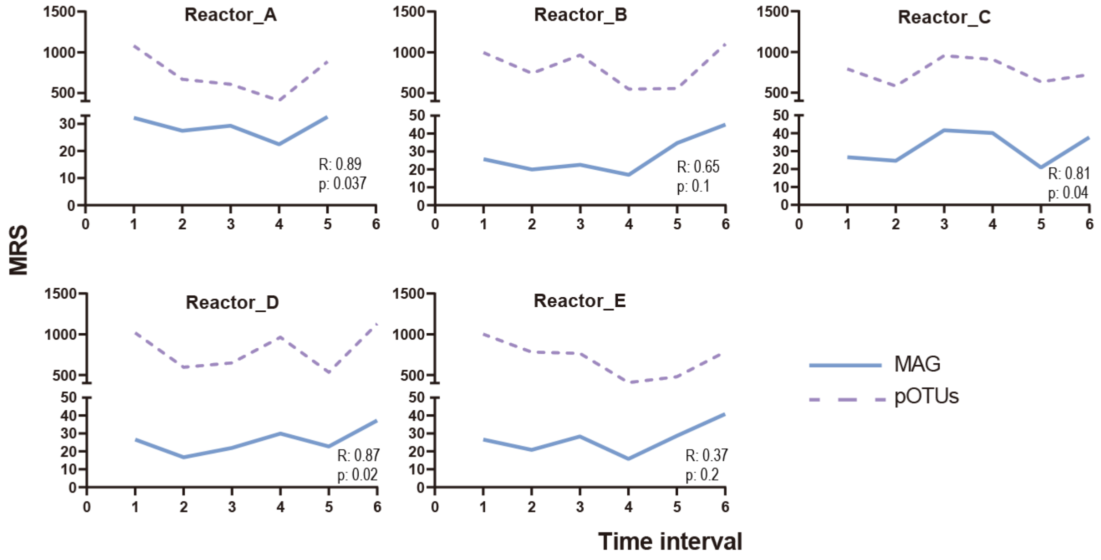

Figure S7 The mean rank shift of pOTUs and MAGs in each community

Mean rank shifts (MRS) represent a temporal analogue of species rank-abundance distributions and indicate the degree of species reordering between two time points. $MRS = \sum_{i=1}^{N}(|R_{i,t+1} - R_{i,t}|)/N$, where N is the number of species in common in both time points, t is the time point and $R_{i,t}$ is the relative rank of species i at time t. Spearman's correlation test was tested between the MRS of bacterial and viral communities.

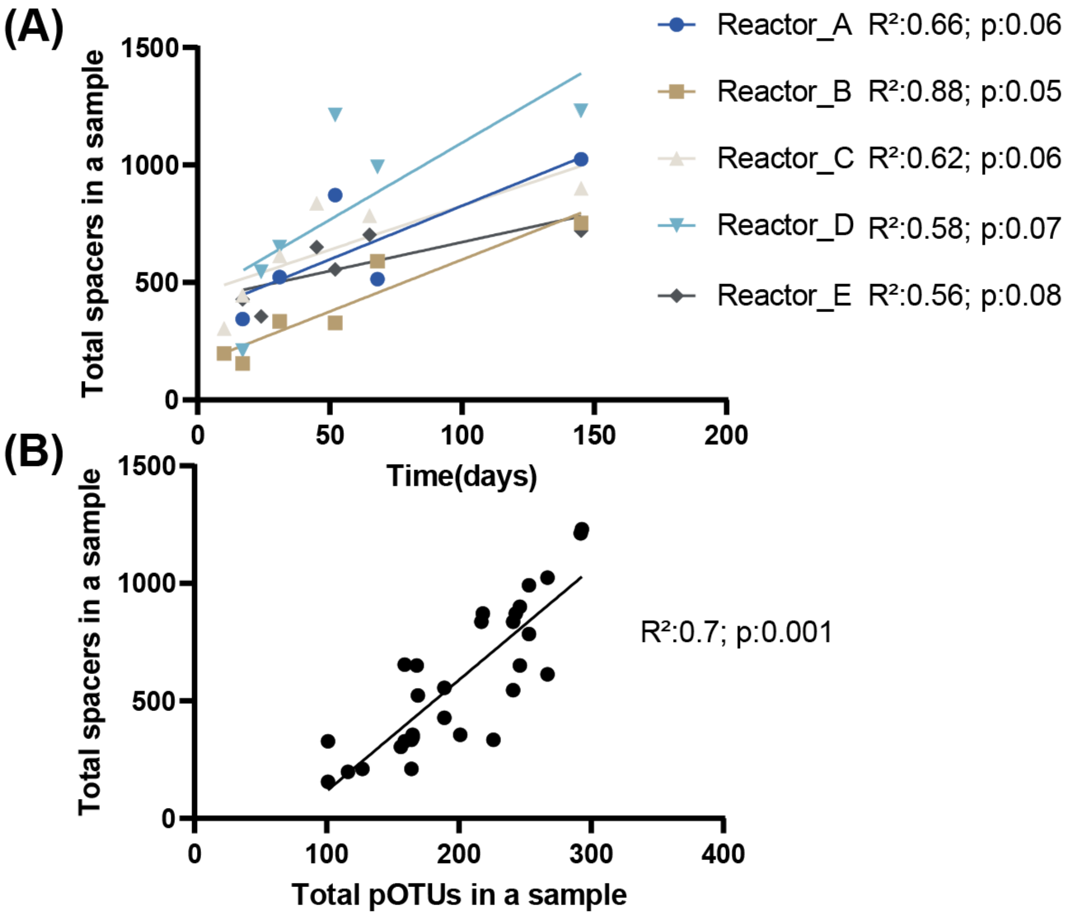

Figure S8 Community-level responses to viral interactions through time as recorded by CRISPR-Cas loci within the microbial community

(A) Spearman’s correlations between the number of spacers in a sample and days. (B) Spearman's correlation between the number of spacers recovered in a sample and the number of pOTUs in a sample.

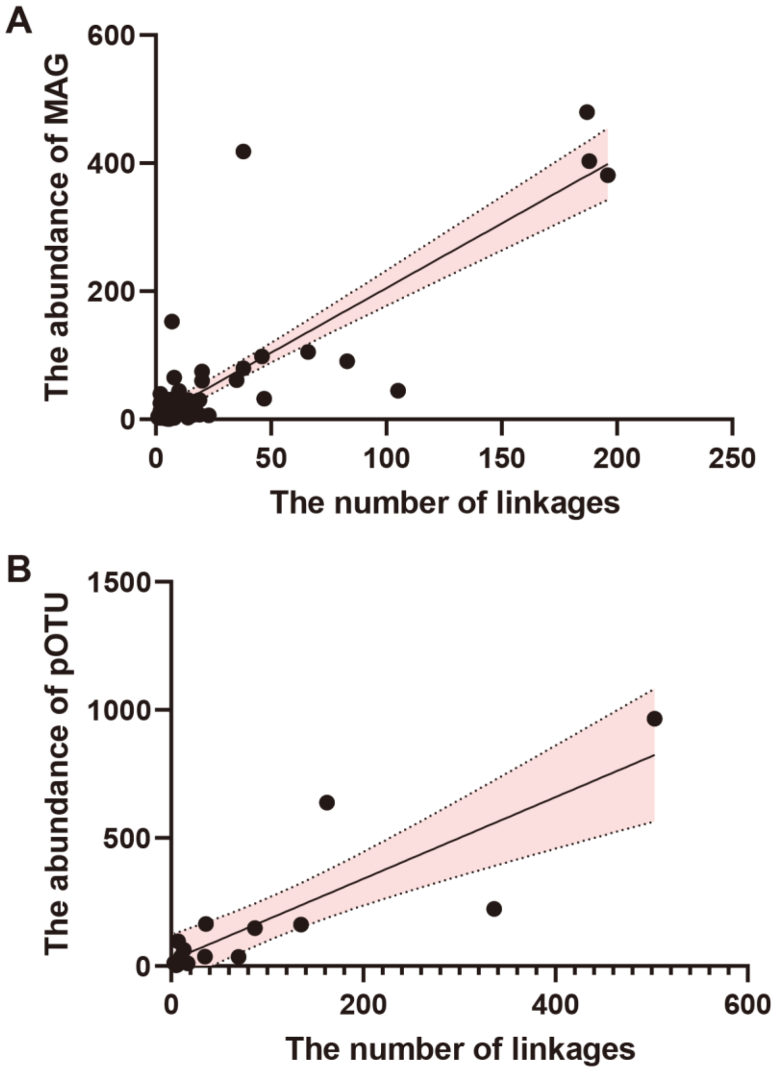

Figure S9 The relationship between the number of linkages and the abundance

(A) The relationship between the number of linkages of prokaryotic MAGs and the abundance of MAGs. (B)The relationship between the number of linkages of pOTUs and the abundance of pOTUs. Red shading represents the 95% confidence region.

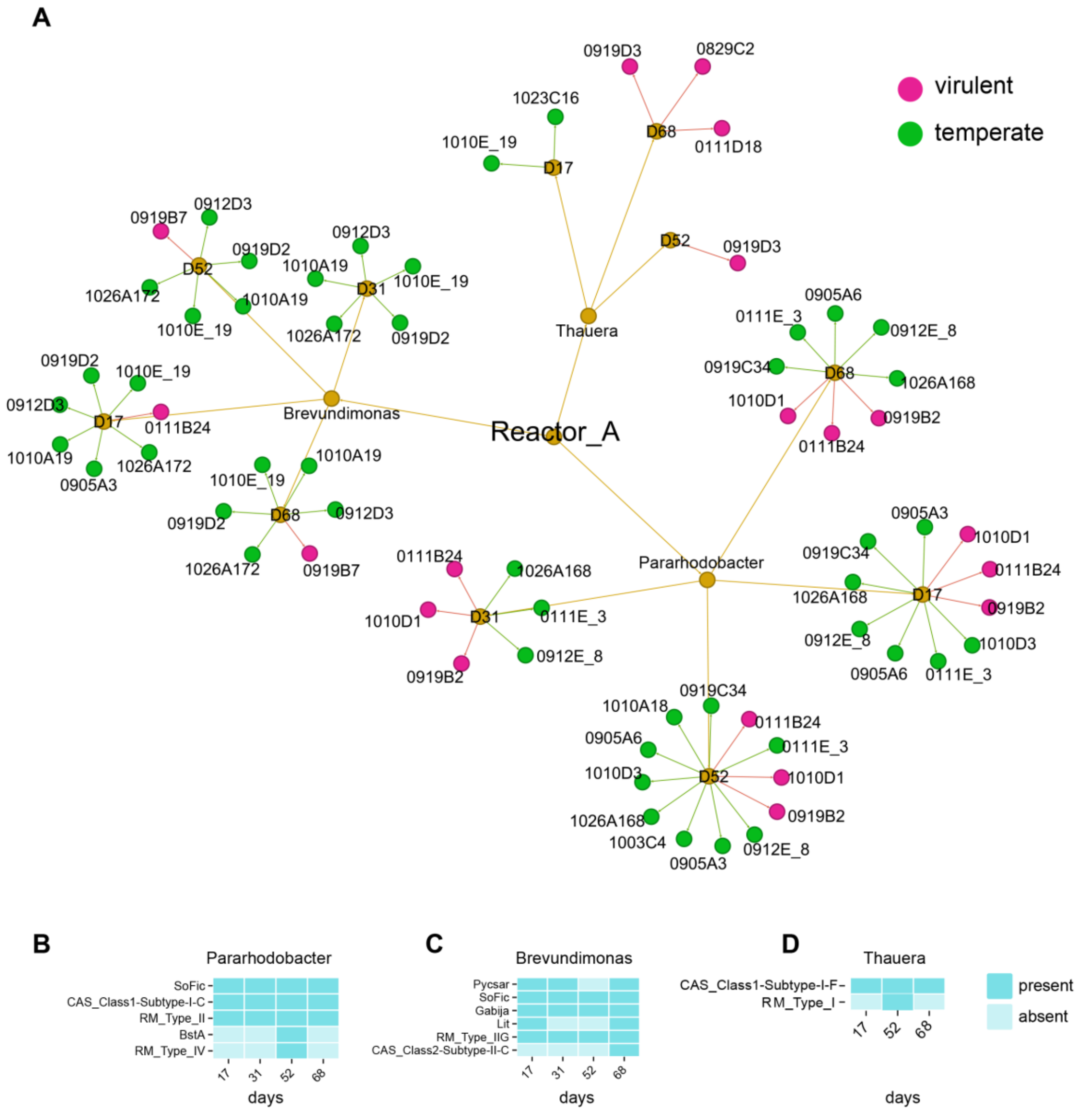

Figure S10 The linkages between phages and hosts on different days and the antiviral systems of hosts (Reactor-A)

(A) The linkages between phages and hosts on different days. The "DX" represents the "X"th day, such as the "D17" represents the 17th day. Red points represent the virulent phages and the green points represent temperate phages. (B) The antiviral systems of *Pararthobacter* on different days. (C) The antiviral systems of *Brevundimonas* on different days. (D) The antiviral systems of *Thauera* on different days. The cyan blocks represent the presence of the antiviral system, while light cyan blocks represent the absence of the antiviral system.

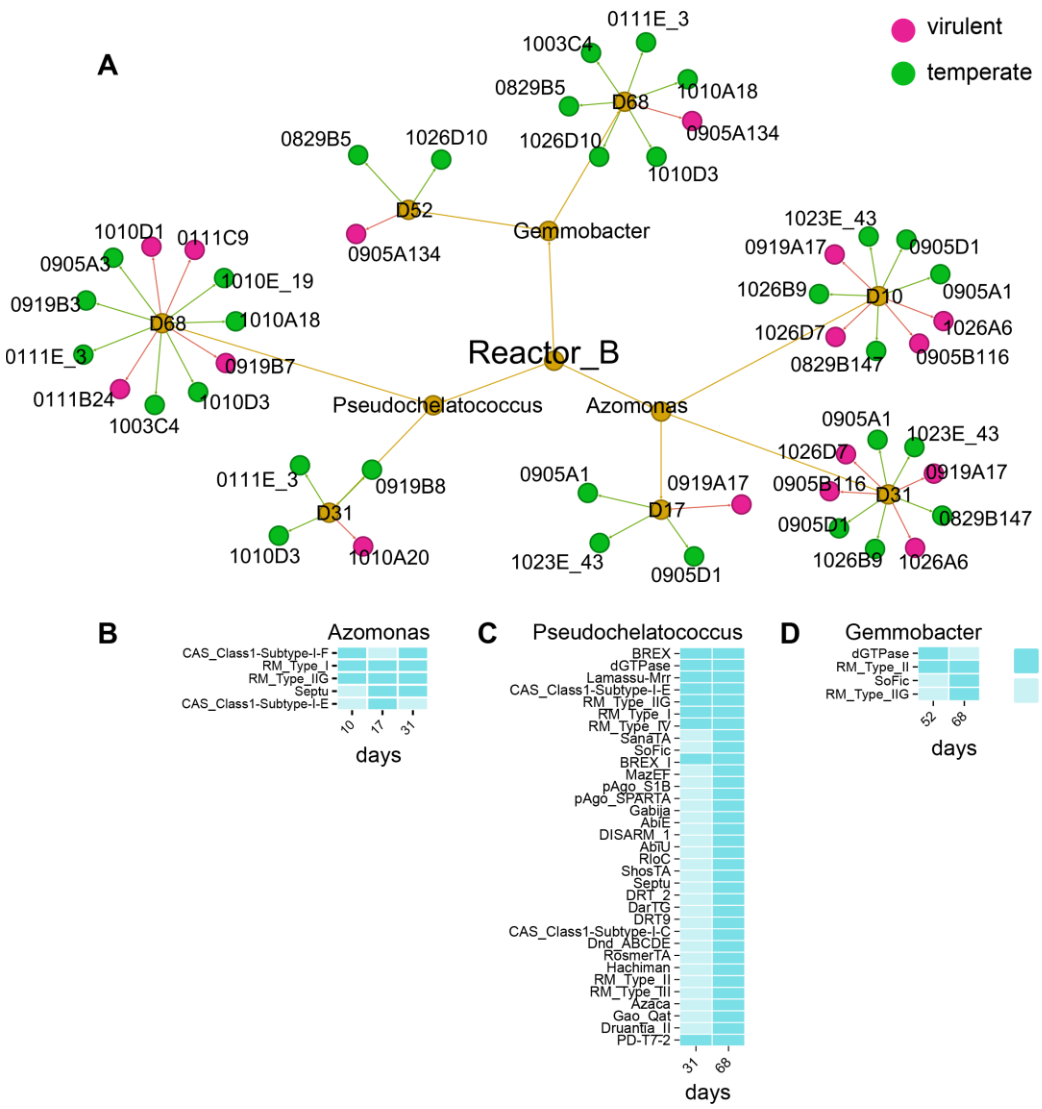

Figure S11 The linkages between phages and hosts on different days and the antiviral systems of hosts (Reactor-B)

(A) The linkages between phage and host on different days. The "DX" represents the "X"th day, such as, the "D17" represents the 17th day. Red points represent the virulent phages and the green points represent temperate phages; (B) The antiviral systems of *Azomonas* on different days. (C) The antiviral systems of *Pseudochelatococcus* on different days. (D) The antiviral systems of *Gemmobacter* on different days. The cyan blocks represent the presence of the antiviral system, while light cyan blocks represent the absence of the antiviral system.

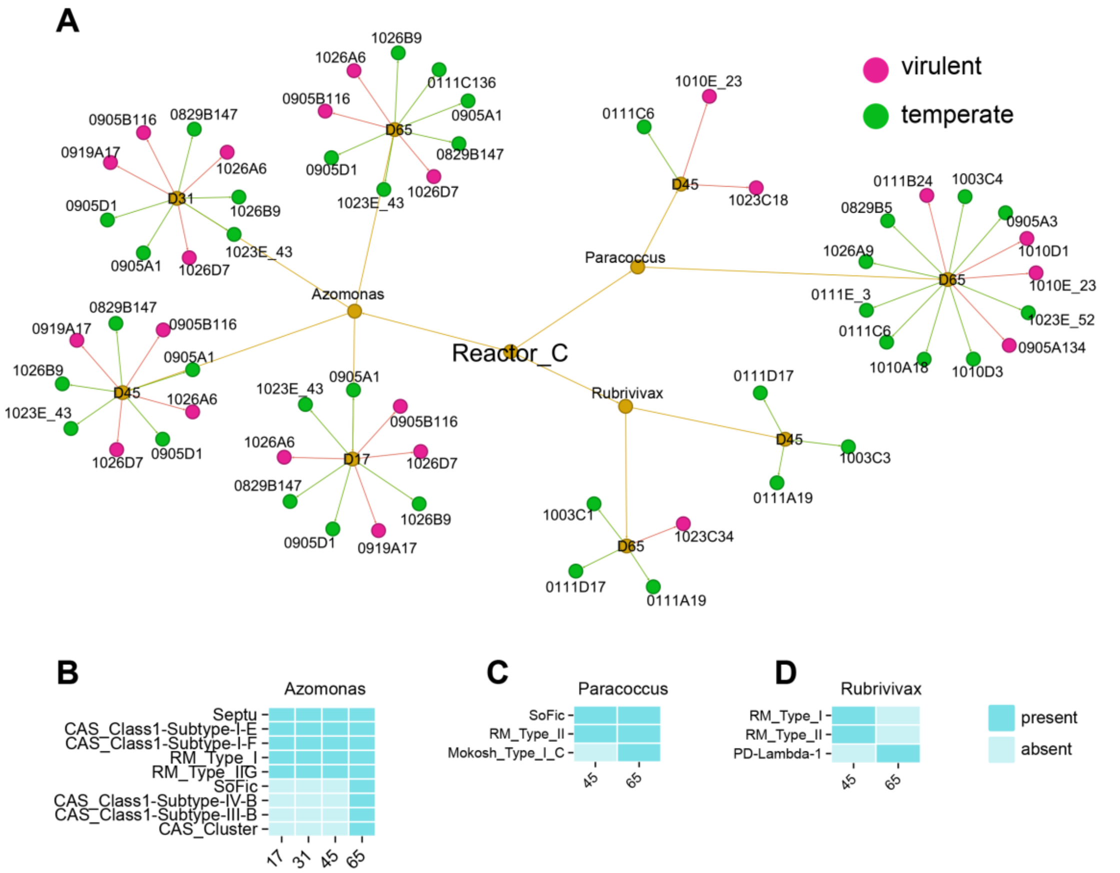

Figure S12 The linkages between phages and hosts on different days and the antiviral systems of hosts (Reactor-C)

(A) The linkages between phage and host on different days. The "DX" represents the "X"th day, such as, the "D17" represents the 17th day. Red points represent the virulent phages and the green points represent temperate phages; (B) The antiviral systems of *Azomonas* on different days. (C) The antiviral systems of *Paracoccus* on different days. (D) The antiviral systems of *Rubrivivax* on different days. The cyan blocks represent the presence of the antiviral system, while light cyan blocks represent the absence of the antiviral system.

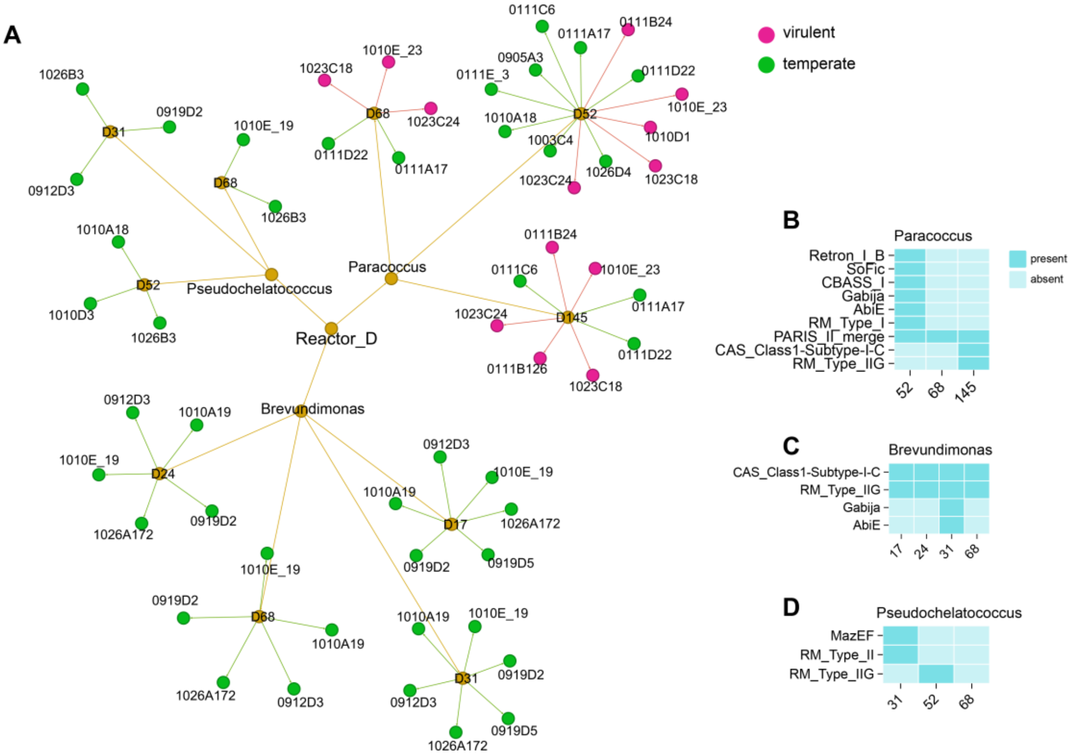

Figure S13 The linkages between phages and hosts on different days and the antiviral systems of hosts (Reactor-D)

(A) The linkages between phage and host on different days. The "DX" represents the "X"th day, such as, the "D17" represents the 17th day. Red points represent the virulent phages and the green points represent temperate phages; (B) The antiviral systems of *Paracoccus* on different days. (C) The antiviral systems of *Brevundimonas* on different days. (D) The antiviral systems of *Pseudochelatococcus* on different days. The cyan blocks represent the presence of the antiviral system, while light cyan blocks represent the absence of the antiviral system.

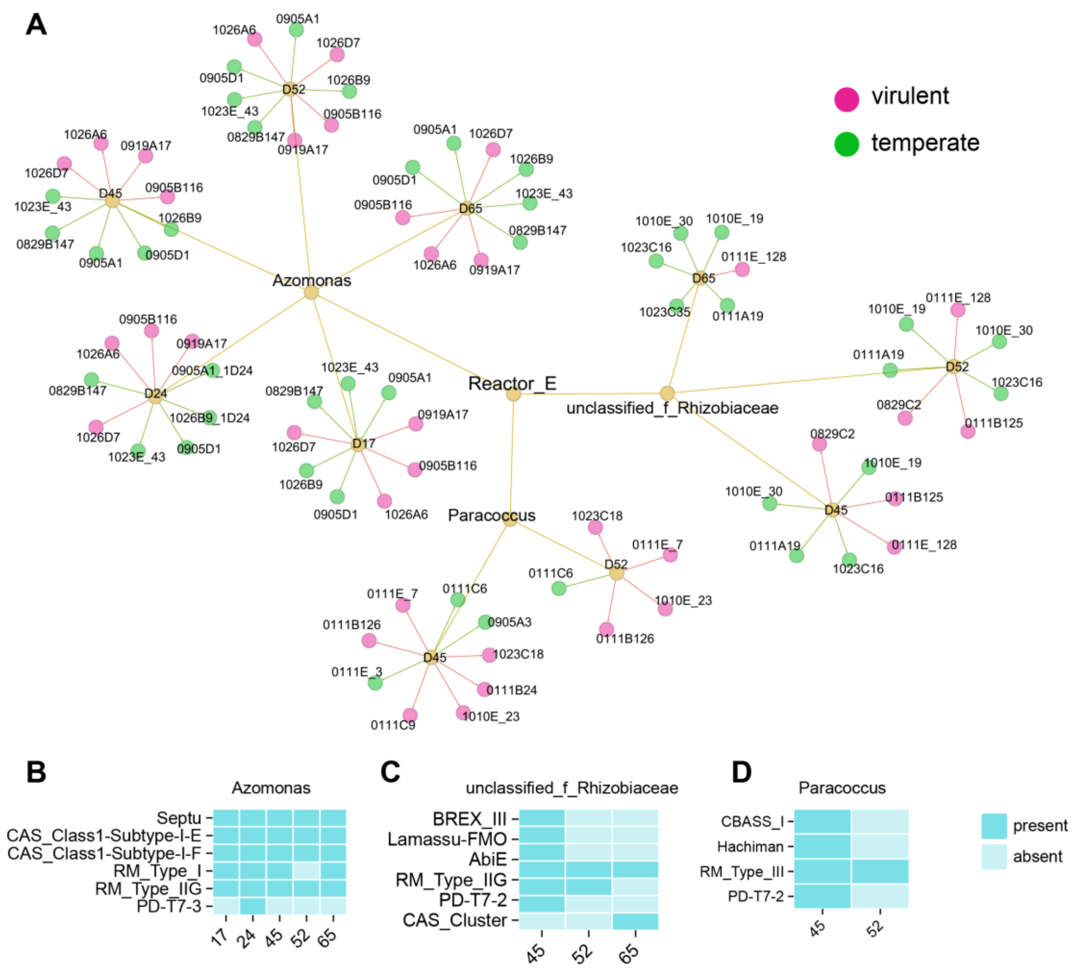

Figure S14 The linkages between phages and hosts on different days and the antiviral systems of hosts (Reactor-E)

(A) The linkages between phage and host on different days. The "DX" represents the "X"th day, such as, the "D17" represents the 17th day. Red points represent the virulent phages and the green points represent temperate phages; (B) The antiviral systems of *Azomonas* on different days. (C) The antiviral systems of *unclassified_f_Rhizobiaceae* (an unclassified genus in family Rhizobiaceae) on different days. (D) The antiviral systems of *Paracoccus* on different days. The cyan blocks represent the presence of the antiviral system, while light cyan blocks represent the absence of the antiviral system.

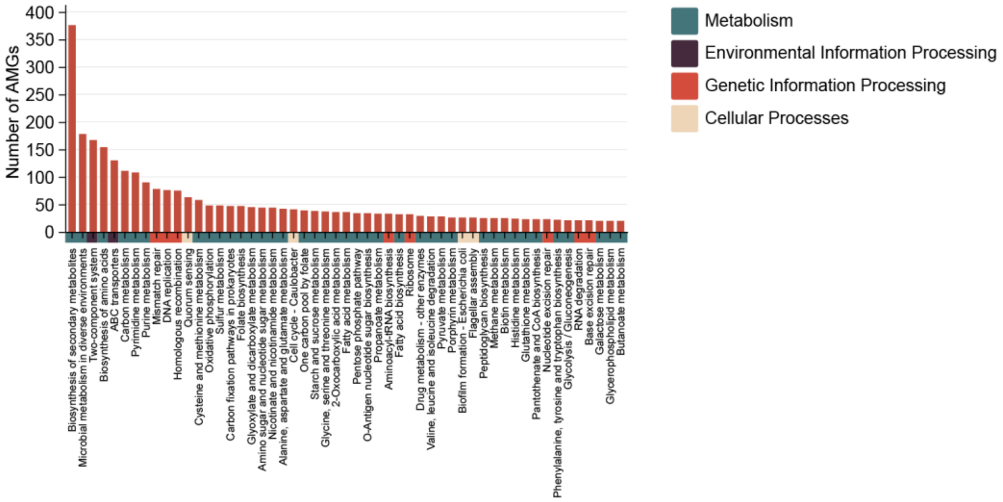

Figure S15 The number of main AMGs in different metabolic pathways

These AMGs were annotated by the KEGG database into different metabolic pathways. These metabolic pathways were classified into four types (metabolism, environmental information processing, genetic information processing and cellular processes).

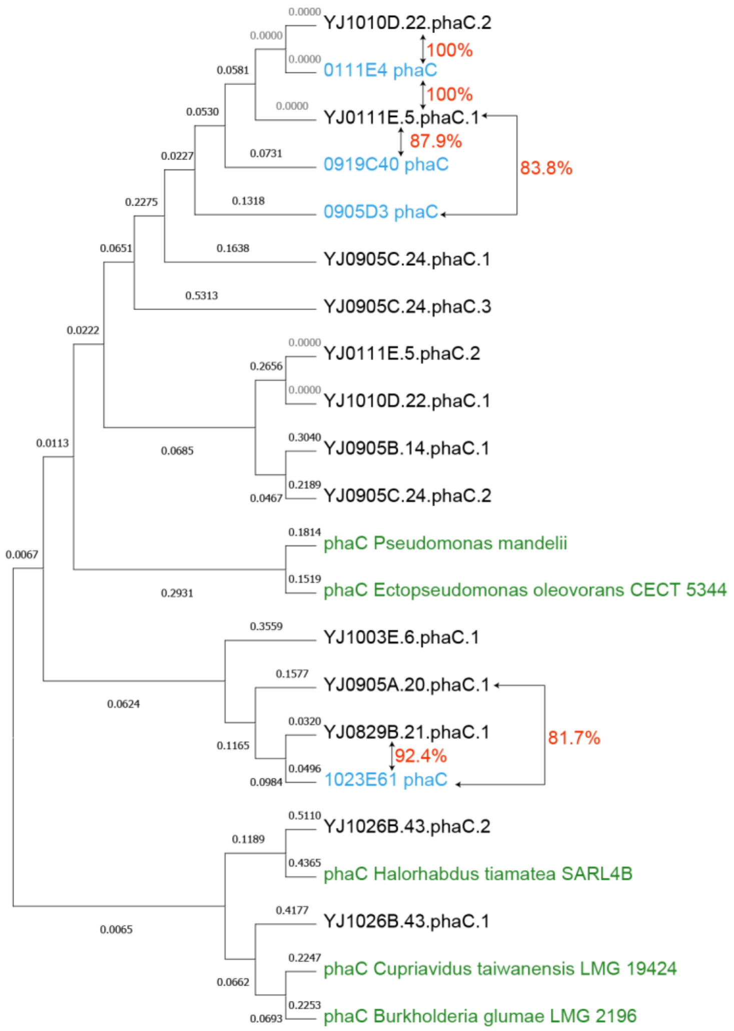

Figure S16 Sequence similarity between phaCs

Red numbers indicate the sequence similarity of AMG pairs (only pairs with identity greater than 75% and e-value less than 1e-20 were listed). The blue font represents AMGs. The black font represents genes in the genome of the prokaryotic hosts. The green font represents reference genes in different organisms. Numerical suffixes (.1; .2; .3; .4) to gene names indicate different copies in the host.

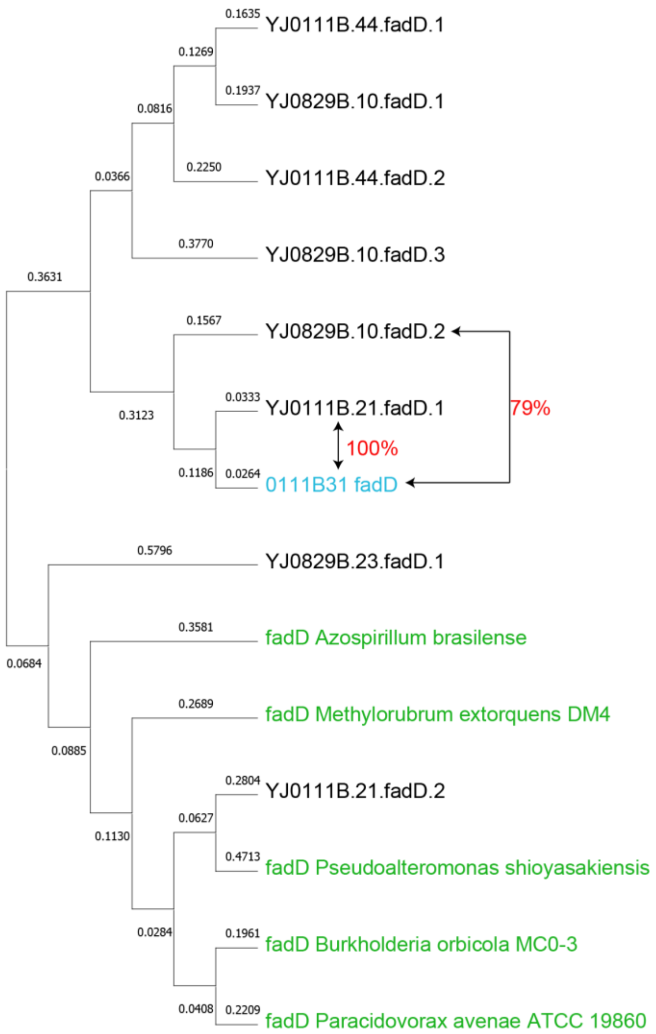

Figure S17 Sequence similarity between fadDs

Red numbers indicate the sequence similarity of AMG pairs (only pairs with identity greater than 75% and e-value less than 1e-20 were listed). The blue font represents AMGs. The black font represents genes in the genome of the prokaryotic hosts. The green font represents reference genes in different organisms. Numerical suffixes (.1; .2; .3; .4) to gene names indicate different copies in the host.

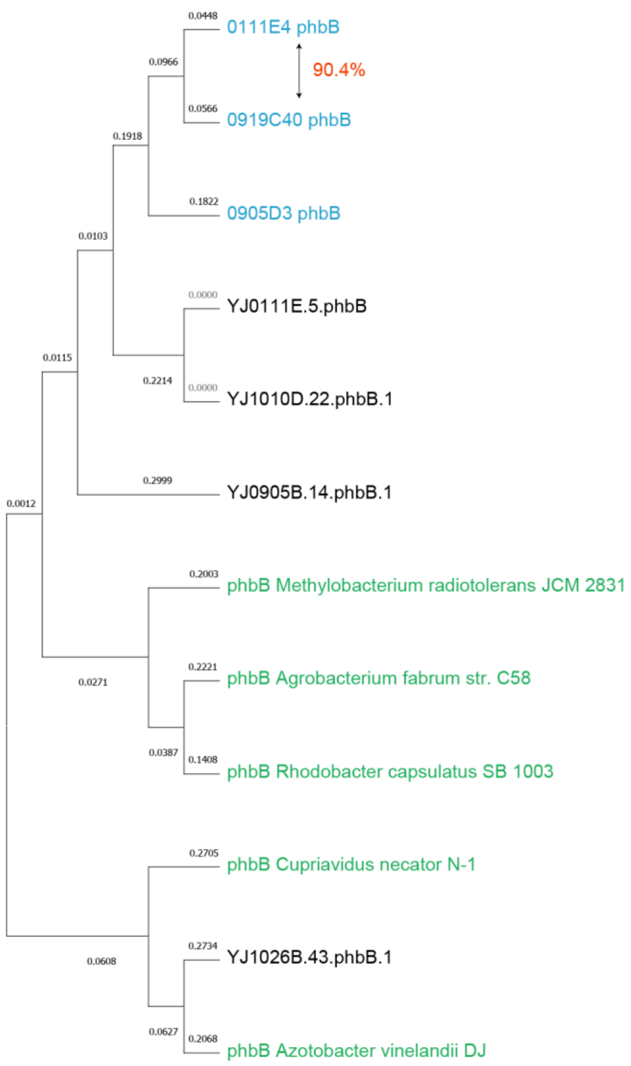

Figure S18 Sequence similarity between phbBs

Red numbers indicate the sequence similarity of AMG pairs (only pairs with identity greater than 75% and e-value less than 1e-20 were listed). The blue font represents AMGs. The black font represents genes in the genome of the prokaryotic hosts. The green font represents reference genes in different organisms. Numerical suffixes (.1; .2; .3; .4) to gene names indicate different copies in the host.

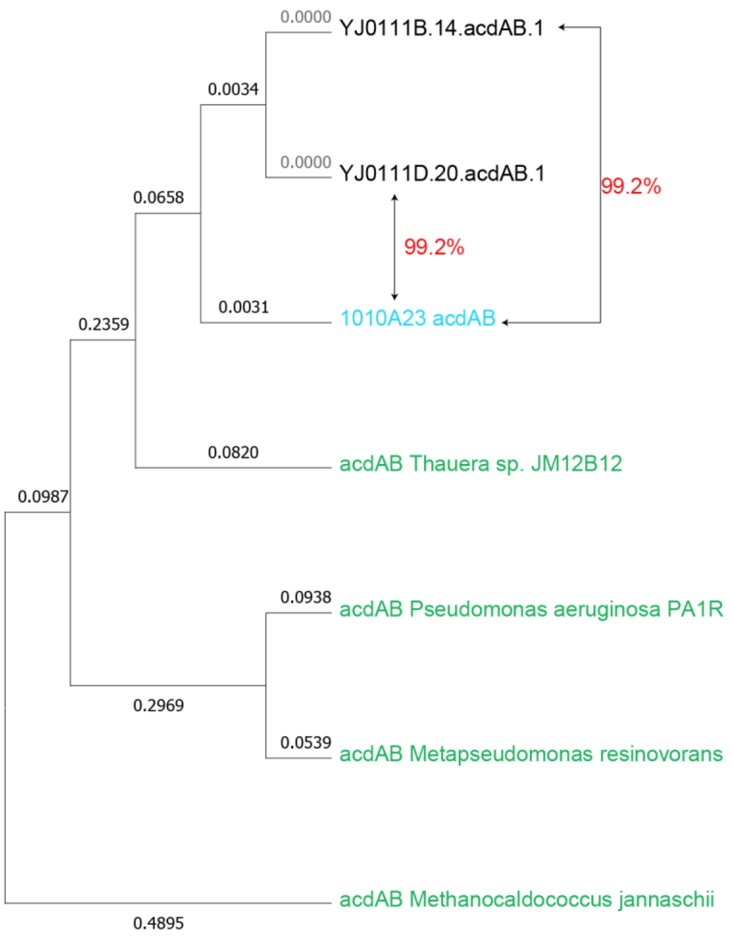

Figure S19 Sequence similarity between acdABs

Red numbers indicate the sequence similarity of AMG pairs (only pairs with identity greater than 75% and e-value less than 1e-20 were listed). The blue font represents AMGs. The black font represents genes in the genome of the prokaryotic hosts. The green font represents reference genes in different organisms. Numerical suffixes (.1; .2; .3; .4) to gene names indicate different copies in the host.

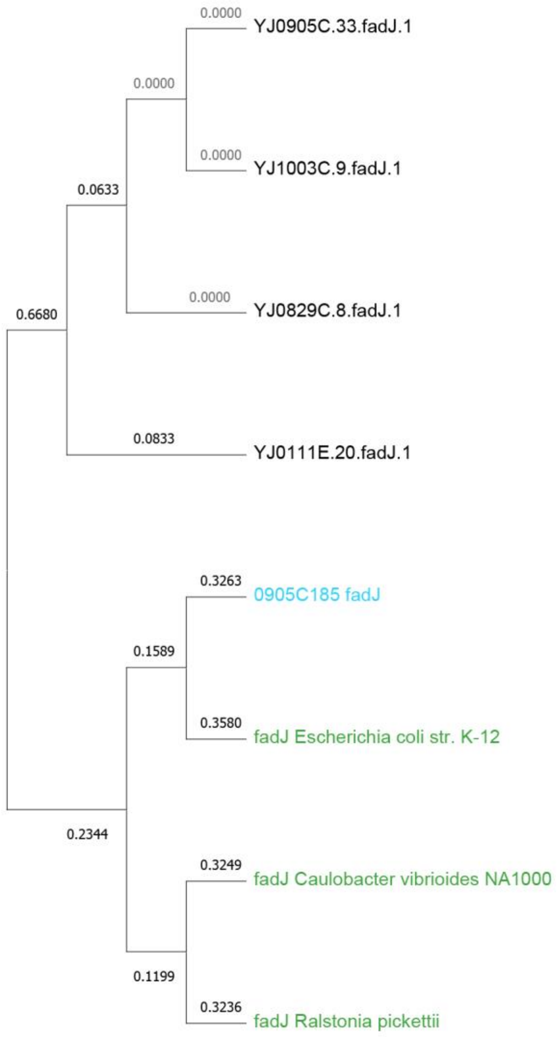

Figure S20 Sequence similarity between fadJs

Red numbers indicate the sequence similarity of AMG pairs (only pairs with identity greater than 75% and e-value less than 1e-20 were listed). The blue font represents AMGs. The black represents genes in the genome of the prokaryotic hosts. The green font represents reference genes in different organisms. Numerical suffixes (.1; .2; .3; .4) to gene names indicate different copies in the host.

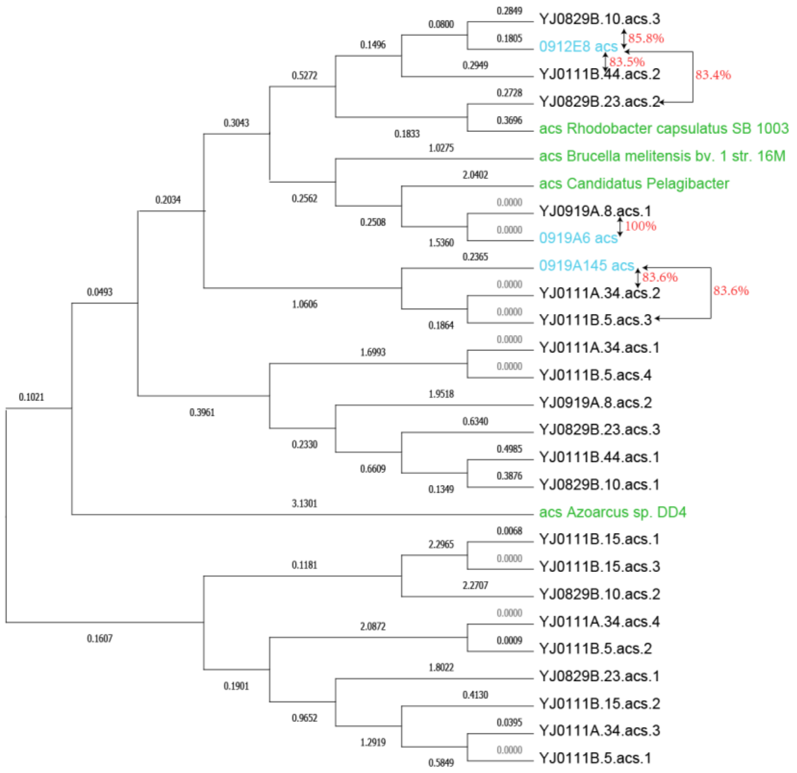

Figure S21 Sequence similarity between acs

Red numbers indicate the sequence similarity of AMG pairs (only pairs with identity greater than 75% and e-value less than 1e-20 were listed). The blue font represents AMGs. The black font represents genes in the genome of the prokaryotic hosts. The green font represents reference genes in different organisms. Numerical suffixes (.1; .2; .3; .4) to gene names indicate different copies in the host.

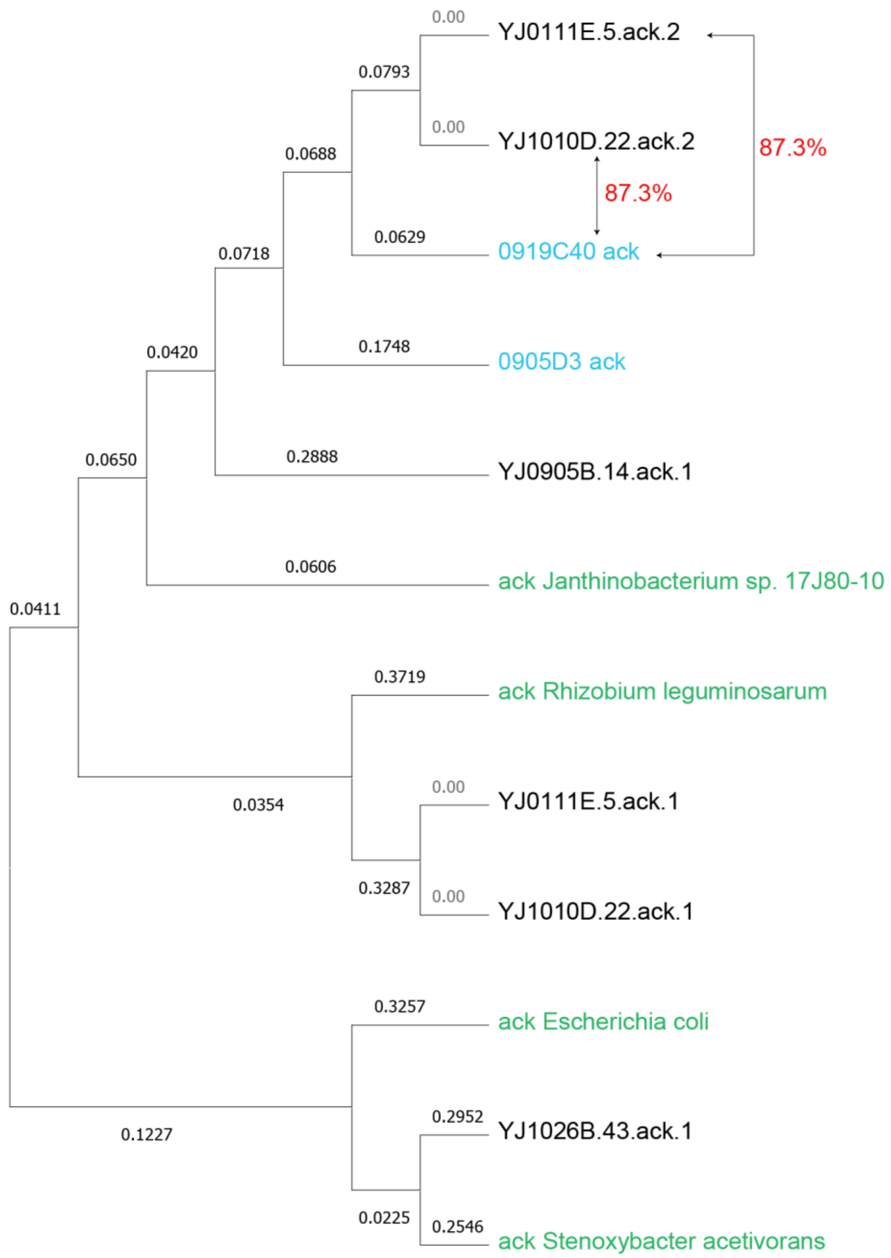

Figure S22 Sequence similarity between ackAs

Red numbers indicate the sequence similarity of AMG pairs (only pairs with identity greater than 75% and e-value less than 1e-20 were listed). The blue font represents AMGs. The black font represents genes in the genome of the prokaryotic hosts. The green font represents reference genes in different organisms. Numerical suffixes (.1; .2; .3; .4) to gene names indicate different copies in the host.

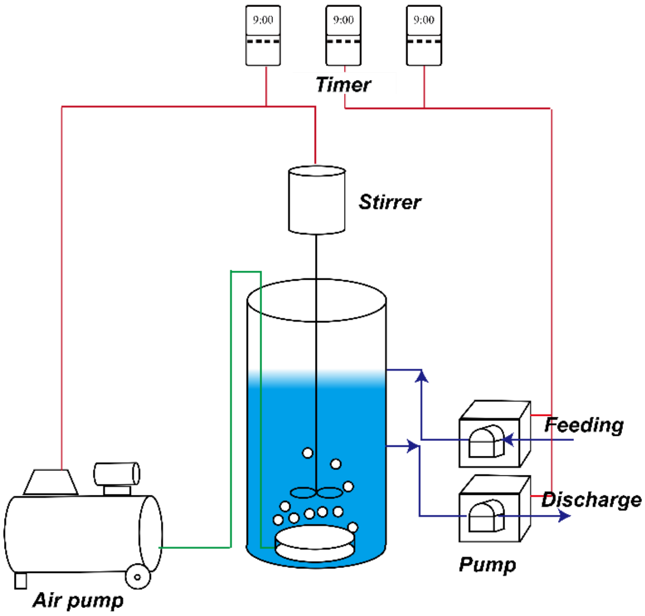

Figure S23 Schematic diagram of SBRs

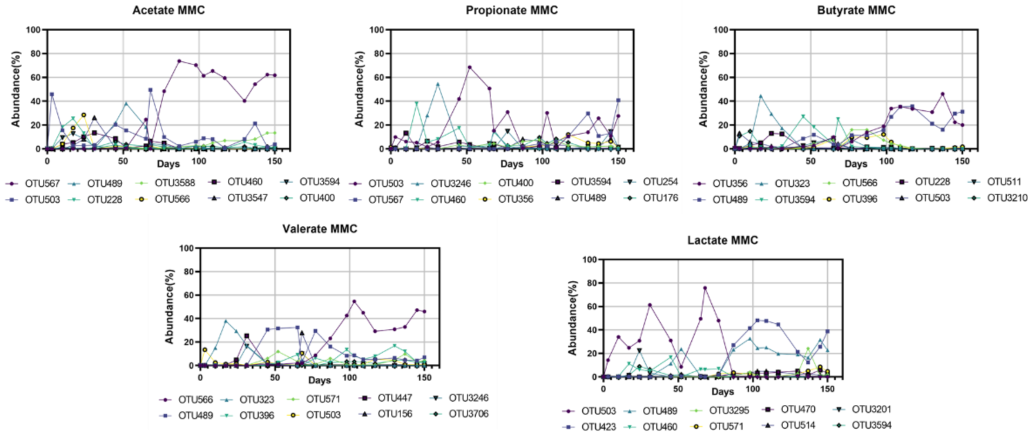

Figure S24 The abundance curve of abundant OTUs in each MMC

Abundance curves of the major OTUs (top ten in average abundance in all samples) in each reactor. Acetate mixed microbial culture (Acetate MMC in reactor A); Propionate mixed microbial culture (Propionate MMC in reactor B); Butyrate mixed microbial culture (Butyrate MMC in reactor C); Valerate mixed microbial culture (Valerate MMC in reactor D); Lactate mixed microbial culture (Lactate MMC in reactor E)
